# Viral FLICE Inhibitory Protein of Rhesus Monkey Rhadinovirus Inhibits Apoptosis by Enhancing Autophagosome Formation

**DOI:** 10.1371/journal.pone.0039438

**Published:** 2012-06-20

**Authors:** Krit Ritthipichai, Yuchen Nan, Ioannis Bossis, Yanjin Zhang

**Affiliations:** 1 Molecular Virology Laboratory, VA-MD Regional College of Veterinary Medicine and Maryland Pathogen Research Institute, University of Maryland, College Park, Maryland, United States of America; 2 Cell Biology Laboratory, VA-MD Regional College of Veterinary Medicine and Maryland Pathogen Research Institute, University of Maryland, College Park, Maryland, United States of America; Hannover Medical School, Germany

## Abstract

Rhesus monkey rhadinovirus (RRV) is a gamma-2 herpesvirus closely related to human herpesvirus 8 (HHV8). RRV encodes viral FLICE inhibitory protein (vFLIP), which has death effector domains. Little is known about RRV vFLIP. This study intended to examine its function in apoptosis. Here we found that RRV vFLIP inhibits apoptosis induced by tumor necrosis factor-α (TNF-α) and cycloheximide. In HeLa cells with vFLIP expression, the cleavage of poly [ADP-ribose] polymerase 1 (PARP-1) and activities of caspase 3, 7, and 9 were much lower than those in controls. Cell viability of HeLa cells with vFLIP expression was significantly higher than control cells after apoptosis induction. However, RRV vFLIP appears unable to induce NF-κB signaling when tested in NF-κB reporter assay. RRV vFLIP was able to enhance cell survival under starved conditions or apoptosis induction. At early time points after apoptosis induction, autophagosome formation was enhanced and LC3-II level was elevated in cells with vFLIP and, when autophagy was blocked with chemical inhibitors, these cells underwent apoptosis. Moreover, RRV latent infection of BJAB B-lymphoblastoid cells protects the cells against apoptosis by enhancing autophagy to maintain cell survival. Knockdown of vFLIP expression in the RRV-infected BJAB cells with siRNA abolished the protection against apoptosis. These results indicate that vFLIP protects cells against apoptosis by enhancing autophagosome formation to extend cell survival. The finding of vFLIP’s inhibition of apoptosis via the autophagy pathway provides insights of vFLIP in RRV pathogenesis.

## Introduction

Rhesus monkey rhadinovirus (RRV) was first found in 1997 in the New England Primate Research Center [1]. It was shown that RRV has close sequence relatedness to Kaposi’s sarcoma-associated herpesvirus (KSHV), a gamma herpesvirus that is associated with Kaposi’s sarcoma, primary effusion lymphoma (PEL) or body cavity based lymphoma (BCBL), and multicentric Castleman’s disease [2], [3], [4]. Two major hindrances for KSHV study are the lack of a permissive lytic system for high yield of infectious virions and an appropriate animal model for the investigation of KSHV pathogenesis [5]. Full length sequences of two different RRV strains were subsequently obtained at the New England Primate Research Center for strain 26–95 [6] and the Oregon Regional Primate Research Center for strain 17577 [6], [7]. The long unique region of the RRV genome is about 130 kb and high overall sequence similarity to KSHV was found in both strains. The RRV genomic organization is collinear with KSHV, with the exception of a few genes encoding homologues of cytokines and interferon regulatory factors. RRV can efficiently replicate without any chemical induction in permissive cell lines like rhesus macaque skin fibroblast cell line (RhF) [8].

High prevalence of antibodies to RRV was found in rhesus monkey colonies at multiple facilities for at least ten years [1], [9], [10]. Experimental infection of rhesus monkeys with RRV led to persistent antibody response and virus detection in lymph nodes, oral mucosa, skin, and peripheral blood mononuclear cells [11], [12]. Co-inoculation of rhesus monkeys with RRV and simian immunodeficiency virus (SIV) resulted in lymphoid hyperplasia comparable to KSHV-associated multicentric Castleman’s disease and has been explored as an animal model for KSHV [12], [13].

Like other herpesviruses, RRV maintains two phases of replication during infection, lytic and latent. RRV ORF71 encodes viral FLICE (FADD-like interleukin-1-converting enzyme)-inhibitory protein (vFLIP), which is expressed during latent phase. The structure of this protein is homologous to cellular FLIP, which contains two death effector domains resembling the amino terminus of caspase 8 [6]. The interaction between death effector domains of FLIP and adaptor protein Fas-associated protein with death domain (*FADD*) can protect cells against apoptosis. In the apoptosis pathway, members of the caspase family of cysteine proteases are key mediators to initiate and execute the apoptotic program [14], [15]. Several caspases (caspase-8, -9, and -10) play upstream “initiator" roles in the process of apoptosis and several others (caspase-3, -6, and -7) are “effector" caspases. The activation of the initiator caspases can be induced by external stimuli from the cell surface in the extrinsic apoptosis pathway or signals originating from inside the cell in the intrinsic apoptosis pathway.

Inhibition of apoptosis is generally observed for vFLIPs of several viruses, such as Herpesvirus Saimiri (HVS), Molluscum Contagiosum virus (MCV), and Sindbis virus (SV) [16], [17], [18]. Besides inhibition of apoptosis, KSHV vFLIP can constitutively activate nuclear factor-κB (NF-κB) pathway through enhancing the degradation of IκB, which allows the RelA/p65 subunit of NF-κB to translocate into the nucleus and promote expression of cellular genes [19]. KSHV vFLIP has also been shown to inhibit autophagy induced by rapamycin, an immunosuppressant and anti-cancer drug [20]. Autophagy is a catabolic process involving the degradation of cytosolic components through the lysosomal machinery.

RRV vFLIP has 174 aa with an expected molecular mass of 20 kDa [6], [7]. There are approximately 40% similarity between KSHV vFLIP and RRV vFLIP at nucleotide level, and 33% identity at amino acid level. But little is known about RRV vFLIP. Here we found that RRV vFLIP inhibits apoptosis and enhances cell survival via enhancing autophagosome formation. The enhancement of cell survival was observed in BJAB B-lymphoblastoid cells latently infected with RRV when the cells were induced to undergo apoptosis. Suppression of vFLIP expression in the RRV-infected BJAB cells with siRNA abolished the protective effect against apoptosis. Our findings provide a novel aspect of vFLIP to inhibit apoptosis by employing autophagosome formation.

## Results

### Cloning and Expression of RRV ORF71 Gene

**Figure 1:**
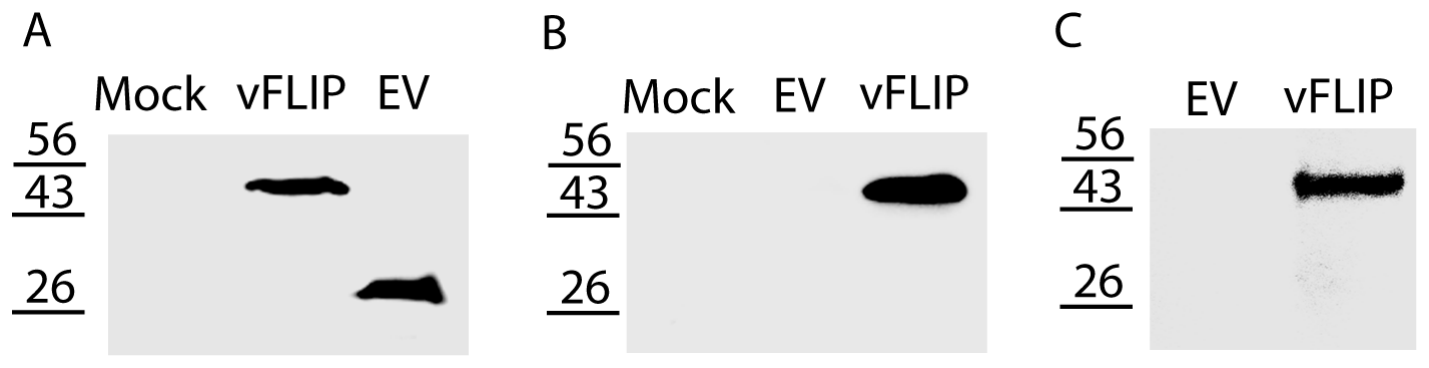
Expression of RRV vFLIP protein is detected by Western blotting. The cells were transfected with VenusN1-vFLIP or empty vector (EV). Lysate of mock-transfected cells was included as a control. A. Detection of vFLIP-Venus fusion protein in HEK293 cells by mouse anti-GFP antibody. Molecular weight markers are indicated on the left of the images. B. Detection of vFLIP-Venus fusion protein in HEK293 cells by rabbit anti-vFLIP antibody. C. Detection of vFLIP-Venus fusion protein in HeLa cells by rabbit anti-vFLIP antibody.

RRV ORF71 was cloned into a VenusN1 vector for expression of vFLIP-Venus fusion protein. HEK293 cells were transfected with either VenusN1-vFLIP or empty vector. The vFLIP-Venus fusion protein was detected by a mouse monoclonal antibody against GFP and the size of the fusion protein was approximately 48 kDa, while cells transfected with the empty vector yielded a band at 27 kDa (Fig. 1A), as expected. Western blot analysis of the cell lysates with rabbit anti-vFLIP antibody detected the 48 kDa fusion protein, while no signal was visible in whole proteins from cells transfected with empty vector (Fig. 1B), demonstrating the specificity of the antibody against vFLIP. These results confirmed the expression of vFLIP fusion protein in the cells transiently transfected. Similarly, the vFLIP fusion protein was detected in HeLa cells transfected with VenusN1-vFLIP (Fig. 1C). The results indicate that vFLIP expression is not cell type-dependent.

### Inhibition of the Apoptosis Signaling Pathway

**Figure 2:**
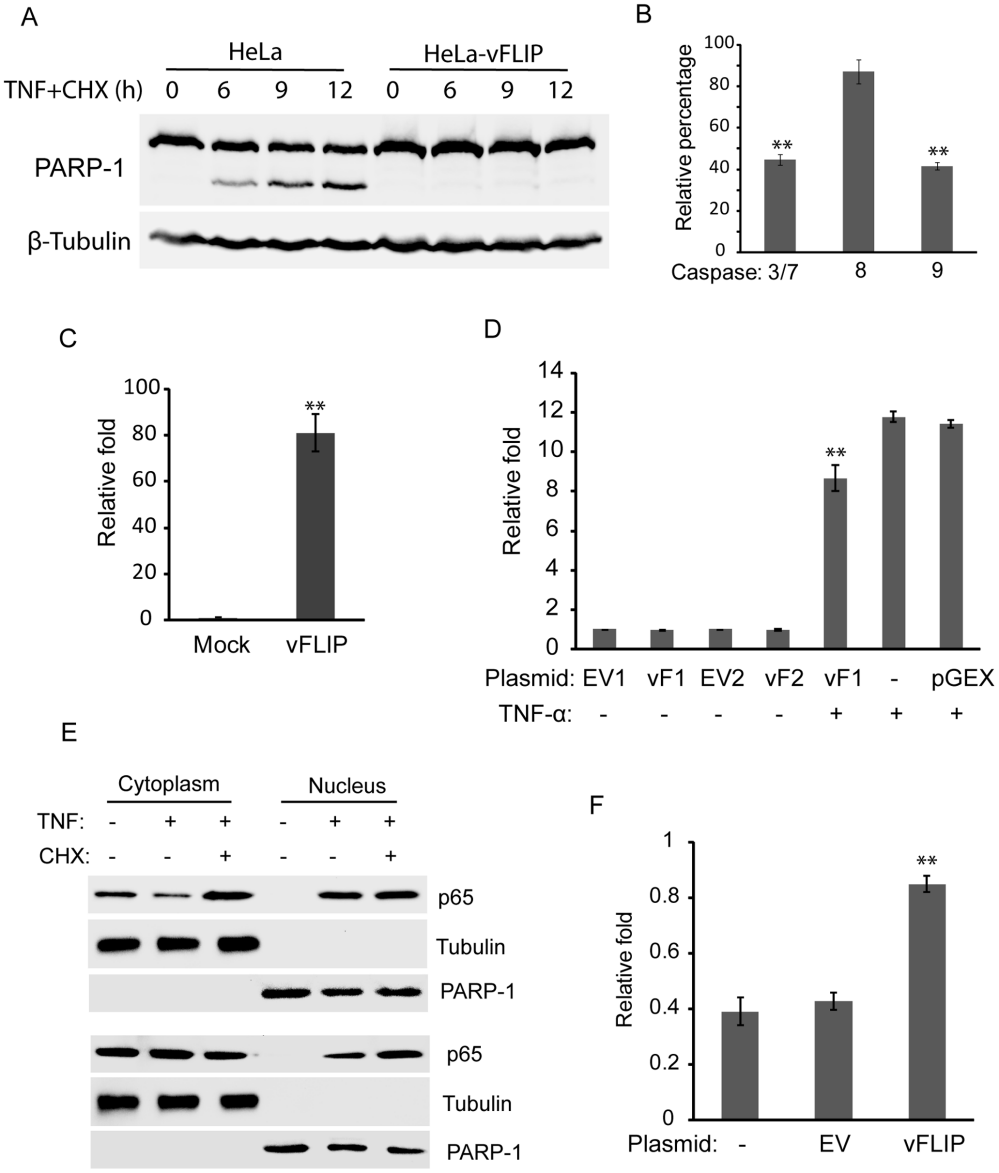
RRV vFLIP inhibits apoptosis in HeLa cells. A. Reduction of PARP-1 cleavage in HeLa cells detected by Western blotting. HeLa and HeLa-vFLIP stable cells were treated with tumor necrosis factor-α (TNF-α) and cycloheximide to induce apoptosis, and harvested at 0, 6, 9, and 12 h after the treatment. PARP-1 cleavage was detected as a marker of apoptosis. Tubulin was detected on the same membrane for loading normalization. B. Reduction of activities of caspase 3/7, 8, and 9. HeLa-vFLIP stable cells and HeLa cells were treated with TNF-α and cycloheximide for 6 h. Relative percentages of caspase activities in comparison with normal HeLa cells are shown. Significant differences between HeLa-vFLIP and HeLa cells are denoted by “**", which indicates *P*<0.01. C. Up-regulation of transcript of MnSOD in HeLa-vFLIP stable cells after apoptosis induction detected by real-time PCR. Relative fold in comparison with control HeLa cells under the same treatment is shown. D. RRV vFLIP is unable to activate the NF-κB luciferase reporter. HeLa cells were transfected with NF-κB reporter plasmid pGL4.32[LUC2P/NF-κB-RE/HYGRO], VenusN1-vFLIP (vF1), VenusC1-vFLIP (vF2), empty vector VenusN1 (EV1), or VenusC1 (EV2). TNF-α was used to activate NF-κB as a positive control. Prokaryotic expression vector pGEX-3X was included as a control. Luciferase signals were measured 4 h after TNF-α addition. Relative folds in comparison with EV1 control are shown. Significant differences between cells with vF1 in the presence or absence of TNF-α induction are denoted by “**". E. RRV vFLIP is unable to induce nuclear translocation of NF-κB subunit p65. HeLa cells (top panel image) and HeLa-vFLIP stable cells (lower panel image) were treated with TNF-α or combination of TNF-α and cycloheximide for 4 h and harvested for fractionation of cytoplasmic and nuclear portions, followed by Western blotting with p65 antibody. Tubulin and PARP-1 were detected on the same membrane to confirm the separation of cytoplasmic and nuclear fractions. F. Extension of cell viability after apoptosis induction. HeLa, HeLa-empty vector (EV) and HeLa-vFLIP cells were tested by CellTiter-Glo Cell Viability Assay at 0 and 38 h after apoptosis induction. Relative folds in comparison with normal HeLa cells at 0 h are shown. Significant differences between HeLa-vFLIP and HeLa-EV cells are denoted by “**".

Since RRV vFLIP contains two death effector domains, we tested its effect on the apoptosis pathway. HeLa and HeLa-vFLIP stable cells were induced to undergo apoptosis with TNF-α and cycloheximide, and were harvested at 0, 6, 9, and 12 h after the treatment. Cleavage of poly(ADP-ribose) polymerase-1 (PARP-1) was assessed. PARP-1 is a nuclear DNA-binding zinc finger protein that is involved in DNA repair [21]. PARP-1 proteolytic cleavage is considered a classic hallmark for apoptosis. The PARP-1 cleavage band at 89 kDa was observed in lysate from control HeLa cells at 6, 9 and 12 h after apoptosis induction, while undetectable in cells expressing vFLIP (Fig. 2A). The results indicate that vFLIP inhibits apoptosis.

Apoptosis induction also leads to cleavage of inactive procaspases to form active caspases. Caspase 8 and 9 represent initiation factors in the extrinsic and intrinsic pathways, respectively, and induce the cleavage of caspase 3 and 7, the executive factors in the apoptosis pathway. To further confirm the effect of vFLIP on apoptosis signaling, we conducted caspase activity assays 6 h after apoptosis induction. Caspase activities of caspase 3/7, 8, and 9 in vFLIP-positive cells were 55%, 13%, and 58%, respectively, lower than those in control HeLa cells (Fig. 2B). The reduction of caspase activities of caspase 3, 7, and 9 in HeLa-vFLIP cells may account for the reduced cleavage of PARP-1.

Reactive oxygen intermediates play a critical role in apoptosis induced by TNF-α and cycloheximide, and overexpression of manganese superoxide dismutase (MnSOD) has been previously shown to prevent apoptosis [22], [23]. Superoxide dismutases (SOD) are a class of enzymes that catalyze the dismutation of superoxide into oxygen and hydrogen peroxide [24]. Three forms of superoxide dismutase are present: SOD1 is located in the cytoplasm, SOD2 in the mitochondria, and SOD3 is extracellular. SOD2 contains manganese in its reactive centre and is also known as MnSOD. The transcript of MnSOD in the HeLa cells was assessed by real time RT-PCR 4 h after apoptosis induction. MnSOD expression was significantly up-regulated to almost 90 folds in HeLa-vFLIP stable cells as compared with control (Fig. 2C). The MnSOD elevation was consistent with the reduction of caspase activity in vFLIP-stable cells.

The upregulation of MnSOD is not due to activation by NF-κB signaling, as vFLIP is unable to activate NF-κB, shown by NF-κB luciferase reporter assay (Fig. 2D). HeLa cells were transfected with a NF-κB reporter plasmid pGL4.32[LUC2P/NF-κB-RE/HYGRO] and VenusN1-vFLIP. VenusC1-vFLIP and an empty vector were also included in the test. TNF-α was included as a positive control to activate NF-κB signaling. The luciferase reporter assay showed that luminescence signal in cells with vFLIP expression was low and similar to cells that were transfected with empty vector, whereas TNF-α treatment of HeLa cells induced 12-fold increase (Fig. 2D). TNF-α treatment of HeLa cells transfected with VenusN1-vFLIP induced 9-fold elevation, which was significantly higher than the cells without treatment. Transfection of HeLa cells with prokaryotic vector pGEX-3X did not affect the NF-κB activation after TNF-α induction. This result indicates that vFLIP is unable to activate NF-κB signaling.

To verify the finding in the NF-κB luciferase reporter assay, subcellular fractionation of HeLa cells was conducted to determine NF-κB subcellular location. After NF-κB is activated by TNF-α, it translocates into the nucleus and activates expression of a myriad of genes. HeLa and HeLa-vFLIP stable cells were either untreated or treated with TNF-α. The addition of TNF-α and cycloheximide to one well was included as a control. The cells were harvested at 4 h after the induction and fractions of the nucleus and cytoplasm were separated. Western blot analysis with antibody against the NF-κB p65 subunit showed that p65 remained in the cytoplasm in cells with stable vFLIP expression, while addition of TNF-α or a combination of TNF-α and cycloheximide led to p65 nuclear translocation (Fig. 2E). The result suggests that vFLIP is unable to cause nuclear translocation of NF-κB. Detection of β-tubulin and PARP-1 in only the cytoplasmic and the nuclear fractions, respectively, demonstrated the successful separation of the two fractions.

The data above showed that RRV vFLIP inhibited the signaling cascade of the apoptosis pathway. To test whether the anti-apoptotic function of vFLIP was sufficient to protect the cells from apoptotic death, we conducted a cell viability assay of the HeLa-vFLIP stable cells at 0 and 38 h after apoptosis induction with TNF-α and cycloheximide. Compared with normal HeLa cells at 0 h, relative cell viability of HeLa-vFLIP stable cells at 38 h after apoptosis induction was 0.85-fold, while that of the control cells was 0.43-fold, similar to 0.39-fold of un-transfected HeLa cells (Fig. 2F). This result indicated that vFLIP expression protected the cells from apoptotic cell death.

### vFLIP Enhances Cell Survival under Starved Condition

**Figure 3:**
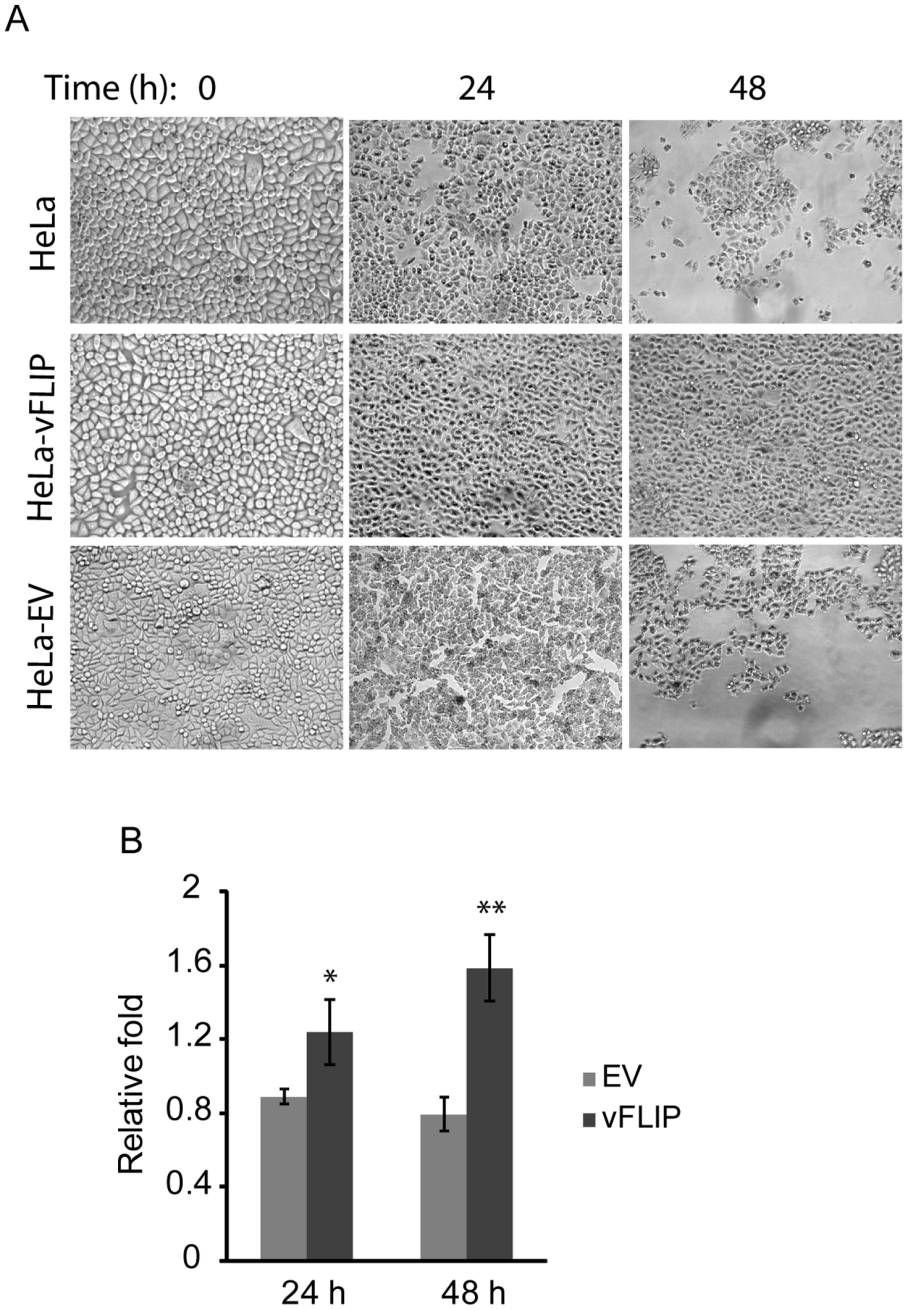
RRV vFLIP enhances survival of HeLa cells under starved condition. A. Bright-field micrographs showing the cells under starvation at 0, 24 and 48 h. Normal HeLa, HeLa-vFLIP stable cells and HeLa-empty vector (EV) cells were starved after culture medium was replaced with Hank’s balanced salt solution (HBSS). B. Cell viability assay of HeLa cells after starvation. The cells were assayed 24 and 48 h after starvation by CellTiter-Glo Cell Viability Assay. Relative folds are shown in comparison with normal HeLa cells at 24, and 48 h, respectively, after normalization of cells at 0 h. Significant differences between HeLa-vFLIP and HeLa-EV cells are denoted by “*" and “**", which indicate *P<0.05* and *P*<0.01, respectively.

Enhanced cell survival is one of the features of tumor cells. To determine whether RRV vFLIP can enhance cell survival under starved condition, we replaced cell culture medium of HeLa, HeLa-vFLIP, and HeLa-VenusN1 cells with Hank’s balanced salt solution (HBSS). These cells were observed at 0, 24, and 48 h after HBSS addition and images were taken under bright field microscopy. HeLa-vFLIP stable cells survived longer than the other cells under starved condition (Fig. 3A). Cell viability of vFLIP-stable cells was 1.24- and 1.58-fold higher than control HeLa cells at 24 and 48 h, respectively, after HBSS addition (Fig. 3B). The cells with empty vector had slightly lower viability levels than control HeLa cells. The result indicated that RRV vFLIP might be involved in autophagy to extend cell survival, since autophagy is a cell survival mechanism to turn over damaged organelles and long-lived proteins in the cytoplasm during starvation.

### vFLIP Enhances Autophagosome Formation in Cells Undergoing Apoptosis Induction

**Figure 4:**
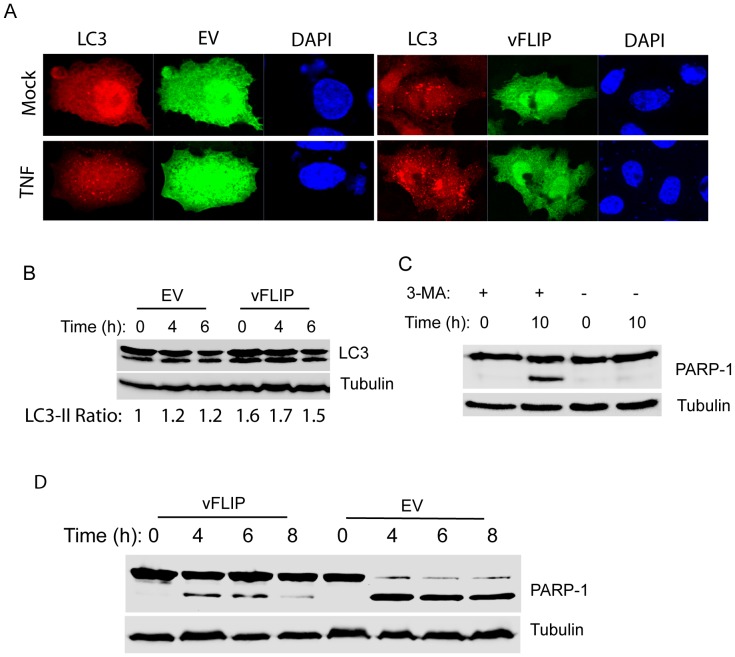
RRV vFLIP enhances autophagosome formation after apoptosis induction. A. RRV vFLIP expression leads to more punctated autophagosomes in HeLa cells after apoptosis induction. HeLa cells were co-transfected with mCherry-LC3 and VenusN1-vFLIP or empty vector (EV), and induced with TNF-α and cycloheximide for 3 h the next day. The cells were observed under confocal fluorescence microscopy. Nuclear DNA was counterstained with DAPI. Mock: no apoptosis induction. TNF: 3 h after addition of TNF-α and cycloheximide. B. Elevation of LC3-II level in cells with vFLIP expression after apoptosis induction. HeLa-vFLIP stable and HeLa cells with EV were transfected with CFP-LC3, and induced with TNF-α and cycloheximide to undergo apoptosis the next day. The cells were harvested 0, 4, and 6 h after the induction for Western blotting of LC3. Tubulin was blotted for normalization. The relative ratios of LC3-II (the lower band of LC3 image) in comparison with HeLa-EV cells 0 h after apoptosis induction are shown below the image. C. RRV vFLIP is unable to inhibit apoptosis when cells are treated with 3-MA. Addition of 3-MA to HeLa-vFLIP stable cells was done 1 h before addition of TNF-α and cycloheximide. The cells were harvested 10 h after TNF-α addition for Western blotting of PARP-1 cleavage. D. Treatment with NH4Cl reduces vFLIP’s capability to inhibit apoptosis. NH4Cl was added to HeLa-vFLIP or HeLa-EV cells at 4, 6, and 8 h after apoptosis induction. The cells without apoptosis induction were included as control.

Autophagy is a dynamic and multi-step process and LC3-II has been widely used as a marker [25], [26]. Microtubule-associated protein 1A/1B-light chain 3 (LC3) is a soluble protein with a ubiquitous distribution in mammalian cells. During autophagy, the cytosolic form of LC3 (LC3-I) is cleaved at its C-terminus and conjugated to phosphatidylethanolamine to form LC3-phosphatidylethanolamine conjugate (LC3-II), which is incorporated into autophagosomal membranes. Thus detecting changes in LC3 lipidation and localization has become a reliable method for monitoring autophagy [27]. HeLa cells were co-transfected with VenusN1-vFLIP plasmid and mCherry-LC3 and, the next day, were induced to undergo apoptosis with TNF-α and cycloheximide. The mCherry-LC3 was used because mCherry is stable in lower pH than CFP and is easier to observe even when autophagosomes are fused with lysosomes [28]. The cells were observed for autophagosome formation 3 h after apoptosis induction. The HeLa cells with vFLIP expression had more punctated autophagosomes after apoptosis induction than cells with empty vector (Fig. 4A). The HeLa cells with vFLIP expression also had visible punctated autophagosomes before apoptosis induction. For Western blot detection of LC3-II in the cells, HeLa-vFLIP stable and HeLa-empty vector cells were transfected with CFP-LC3 plasmid and, the next day, were induced to undergo apoptosis. The cells were harvested for western blotting at 0, 4, and 6 h after apoptosis induction. Densitometry analysis showed that LC3-II in HeLa-vFLIP stable cells at 0, 4, and 6 h after apoptosis induction were 1.6-, 1.7- and 1.5-fold higher than that in HeLa-empty vector cells at 0 h (Fig. 4B). At 4 and 6 h after apoptosis induction, the LC3-II in HeLa-empty vector cells increased to 1.2-fold higher than 0 h.

To determine whether autophagy plays a role in vFLIP’s inhibition of apoptosis, two experiments were performed to inhibit autophagy pathway at two critical steps; autophagosome formation and degradation of autophagosomes in lysosomes. HeLa-vFLIP stable cells were treated with 3-MA for one hour before apoptotic induction to inhibit autophagosome formation. The cells were harvested at 10 h after apoptotic induction for Western blotting. The band of cleaved PARP-1 at 89 kDa was observed in the cells treated with 3-MA, but could not be seen in the mock-treated control (Fig. 4C). The result indicated that after 3-MA treatment of the cells, vFLIP could no longer protect the cells from apoptosis induction. This suggested that autophagosome formation was needed for the anti-apoptotic function of RRV vFLIP, which was consistent with the observation of a significant increase of punctated autophagosomes after apoptosis induction.

Further testing was conducted to determine whether final degradation of autophagic cargo inside autophagolysosomes had any effect on vFLIP’s inhibition of apoptosis. HeLa-vFLIP stable cells were treated with ammonium chloride at 4, 6, and 8 h after apoptosis induction to prevent acidification in lysosomes and harvested for Western blot analysis. In HeLa-vFLIP stable cells, the band of cleaved PARP-1 at 89 kDa was observed at 4 and 6 h, and became weaker at 8 h, while in HeLa cells with empty vector, a strong band of PARP-1 at 89 kDa was observed at all three time points (Fig. 4D). This result indicated that inhibition of autophagolysosomes at 4 and 6 h after apoptosis induction had an inhibitory effect on vFLIP’s anti-apoptotic function.

### Latent RRV Infection of BJAB Cells Protects the Cells Against Apoptosis via Autophagy, and Suppression of vFLIP Expression Abolishes the Protection

**Figure 5:**
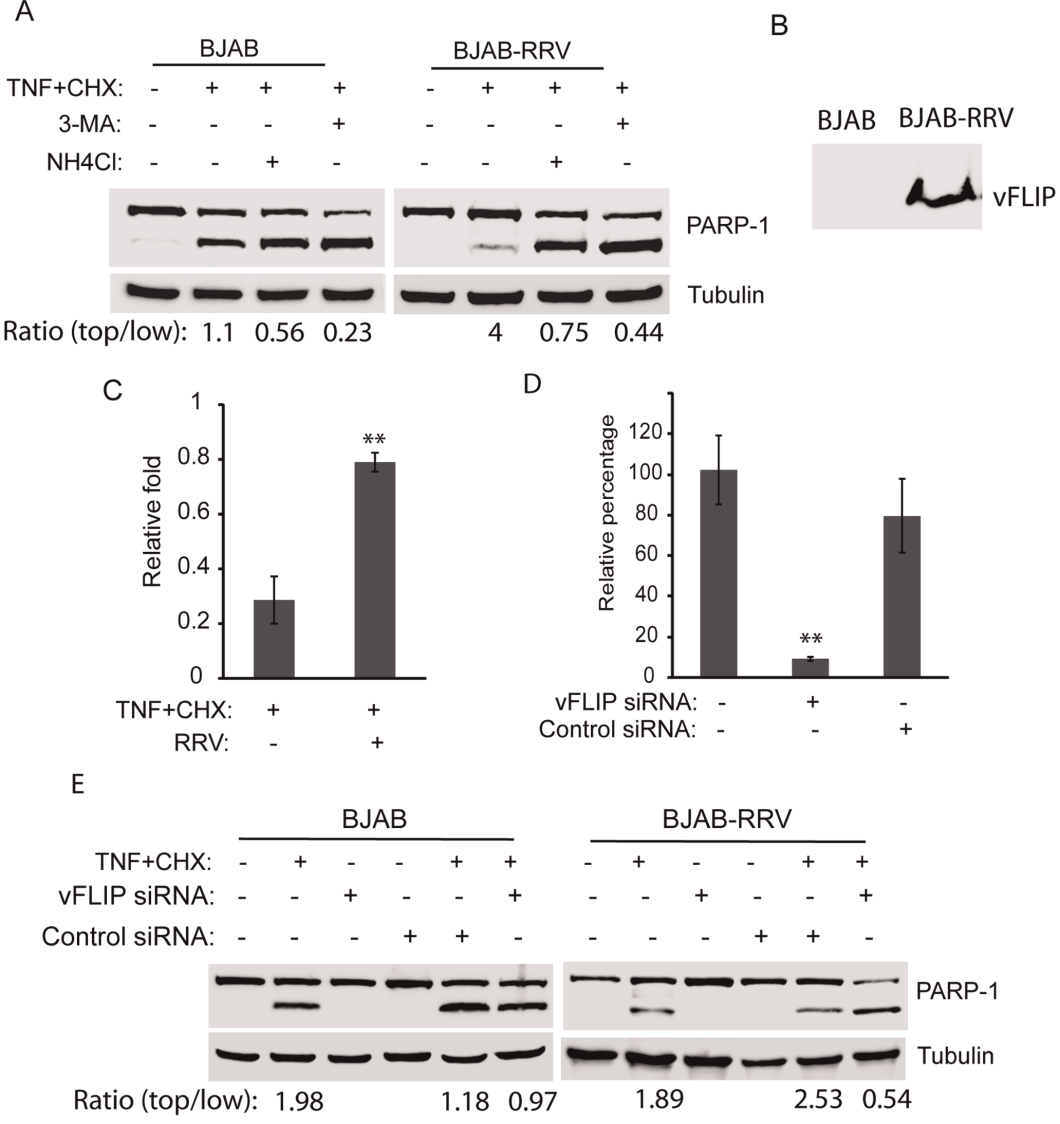
RRV latent infection of BJAB cells protects the cells from apoptosis. A. RRV latent infection of BJAB cells protects the cells against apoptosis and inhibition of autophagy abolishes the protective effect. BJAB cells latently infected with RRV (BJAB-RRV) were either untreated, treated with 3-MA for 3 h prior apoptosis induction by TNF-α and cycloheximide, or treated with ammonium chloride at the same time of the apoptosis induction. The cells were harvested 2 h post-apoptosis induction for Western blotting of PARP-1 cleavage. Similar treatment of uninfected BJAB cells was included as a control. The ratio of top PARP-1 band to low band is shown under the image. B. Detection of vFLIP in RRV-infected BJAB cells by Western blotting with rabbit anti-vFLIP antibody. C. Cell viability assay of BJAB and BJAB-RRV cells 3 h after apoptosis induction. Relative folds in comparison with uninfected BJAB cells at 0 h are shown. Significant differences between BJAB and BJAB-RRV cells after apoptosis induction are denoted by “**", which indicates *P*<0.01. D. Suppression vFLIP expression in RRV-infected BJAB cells by siRNA. BJAB cells latently infected with RRV were transfected with a siRNA against vFLIP. An irrelevant siRNA was included as a control. Real-time RT-PCR was conducted to assess vFLIP transcript level. Relative percentages in comparison with mock-treated control are shown. Significant differences between siRNA-treated and mock-treated BJAB-RRV cells are denoted by “**". E. Suppression of RRV vFLIP gene expression in BJAB-RRV cells leads to loss of the capability against apoptosis. BJAB cells latently infected with RRV were transfected with siRNA against vFLIP 15 h before apoptosis induction. Treatment of uninfected BJAB cells was included as a control. The cells were harvested 2 h after treatment with TNF-α and cycloheximide for Western blotting of PARP-1 cleavage. The ratios of top PARP-1 band to low band are shown under the image.

Although several cell lines can be infected with RRV, the main target cells of RRV latent infection *in vivo* are B cells [9]. BJAB cells were used to examine vFLIP’s role in RRV-infected cells since vFLIP is a latent protein that could play a role during the latent phase of RRV infection. BJAB cells were infected with RRV at a multiplicity of infection (MOI) of 2 TCID_50_ per cell and were maintained for two weeks in culture. BJAB cells latently infected with RRV (BJAB-RRV) and normal BJAB cells were induced to undergo apoptosis and were harvested for Western blot analysis at 2 h after the treatment. The cleaved band of PARP-1 at 89 kDa was strong in BJAB, but much weaker in BJAB-RRV cells (lane 2 in both images of Fig. 5A). The ratio of top band to low band of PARP-1 for BJAB-RRV cells was 4, while 1.1 for BJAB cells. To test whether autophagy was needed for the inhibition of apoptosis in BJAB-RRV, cells were treated with 3-MA for 3 hours prior to apoptosis induction, or ammonium chloride at the same time as apoptosis induction. PARP-1 cleavage was highly increased in BJAB-RRV when treated with either 3-MA or ammonium chloride (lane 3 and 4 in the second image of Fig. 5A). The ratio of top band to low band of PARP-1 in BJAB-RRV when treated with ammonium chloride or 3-MA was reduced to 0.75 and 0.44, respectively. These treatments also increased PARP-1 cleavage in normal BJAB cells (lane 3 and 4 in the first image of Fig. 5A), as expected. This result indicated that the anti-apoptotic function coupled with autophagosome formation in RRV-infected BJAB cells corroborated with the data from HeLa cells stably expressing vFLIP. The vFLIP protein was detected in RRV-infected BJAB cells (Fig. 5B). The cell viability assay showed that viability of BJAB-RRV cells at 3 h after apoptosis induction was 0.79-fold of the cells at 0 h, while viability of BJAB cells 3 h after apoptosis induction was 0.29-fold of the cells at 0 h (Fig. 5C). The result indicated that RRV infection significantly extended the survival of BJAB cells after apoptosis induction, which was consistent with the results of PARP-1 cleavage.

To further confirm that the inhibition of apoptosis via the autophagy pathway in RRV-infected BJAB cells was due to vFLIP expression, BJAB-RRV cells were treated with a siRNA against vFLIP to knockdown vFLIP expression in the cells. An irrelevant siRNA was included as a control. To test if the siRNA against vFLIP could knockdown vFLIP expression in BJAB-RRV cells, real-time RT-PCR was conducted. The results showed that the treatment of BJAB-RRV cells with siRNA against vFLIP reduced vFLIP mRNA to 10% in comparison with mock-treated control, while vFLIP mRNA level in cells with control siRNA had no significant difference from mock-treated control (Fig. 5D). The result indicated that the siRNA to vFLIP effectively suppressed the vFLIP expression in BJAB cells latently infected by RRV.

The BJAB-RRV cells were induced to undergo apoptosis the next day after siRNA transfection. Western blot analysis showed that PARP-1 cleavage was increased in BJAB-RRV cells after treatment with vFLIP siRNA in comparison with the control siRNA (lane 6 of both images in Fig. 5E). The ratio of top to low band of PARP-1 in BJAB-RRV cells when treated with vFLIP siRNA was reduced to 0.54, while 2.53 for the cells with control siRNA. The result indicated that vFLIP was needed for apoptosis inhibition. Treatment of normal BJAB cells with both siRNAs led to a slight increase in PARP-1 cleavage after apoptosis induction. The siRNA treatment of the BJAB cells without apoptosis induction had no effect on PARP-1 level. This result suggested that vFLIP could inhibit apoptosis via the autophagy pathway in RRV-infected cells.

## Discussion

Several γ-herpesviruses contain vFLIP genes. However, not all of the vFLIPs have similar functions. In this study, we found RRV vFLIP is able to inhibit apoptosis via enhancing autophagosome formation. Unlike KSHV vFLIP, RRV vFLIP cannot activate NF-κB. A unique motif, PYQLT, was found in the second death effector domain of KSHV vFLIP, and has the function of NF-kB activation by directly binding to TNF receptor associated factor 2 (TRAF2) [29]. But the TRAF-interacting motif is not available in RRV vFLIP, which may be the reason for failing to activate NF-kB pathway.

A combination of TNF-α and cycloheximide was used to induce apoptosis as either one of them was unable to induce apoptosis at the concentrations used in this work. The cycloheximide sensitizes the cells to undergo apoptosis induced by TNF-α [30], [31]. The activation of caspase 9 indicates that the intrinsic pathway was activated by the apoptosis induction. We speculate that the activation of the intrinsic pathway might be due to the combination of the two compounds in inducing cross talk from extrinsic pathway or the detection time after the induction. The second point is less likely as we tested caspase activity in several time points after the induction and a similar trend was observed. Our finding of up-regulation of MnSOD in HeLa-vFLIP stable cells is consistent with the activation of the intrinsic pathway. MnSOD contributes to suppression of apoptosis through reducing accumulation of intracellular superoxide to enhance cell survival [32].

MnSOD gene expression in HeLa cells with RRV vFLIP expression was elevated in the absence of NF-κB activation. This finding suggests that the NF-κB pathway may not be the only factor to control MnSOD expression, but other transcription factors could also be involved in the regulation of this gene as well. It was reported that p53 responds to physiological stress by stimulating redox-controlling genes to reduce the ROS level [33]. The increase of the MnSOD transcript in HeLa cells with vFLIP expression suggests that RRV vFLIP might employ other transcription factors.

Expression of vFLIP in HeLa cells enhanced cell survival under starved condition. Since nutrient deprivation induces autophagy [34], extension of cell survival of HeLa-vFLIP cells indicates that vFLIP suppresses autophagy. Autophagy is a multiple-step process that begins with the formation of autophagosome-cytoplasmic vesicles that have a double membrane and contain cytoplasmic cargo, proceeds with fusion of autophagosomes with lysosomes to become autophagolysosomes, and ends with degradation of the contents in the autophagolysosomes. Enhancement of cell survival under starved condition in HeLa-vFLIP stable cells prompted us to determine autophagy before and after apoptosis induction. Interestingly, we found that autophagosome formation was increased in HeLa-vFLIP stable cells at early time points after apoptosis induction. When autophagy was inhibited at either early autophagosome formation by 3-MA or late autophagosome degradation by ammonium chloride, vFLIP could no longer protect the cells against apoptosis. The addition of ammonium chloride at 8 h after apoptosis induction to inhibit autophagosome degradation had less effect on the function of RRV vFLIP in inhibition of apoptosis than earlier time points. The apoptosis induction in HeLa cells is much more efficient in the presence of ammonium chloride. Our result suggests that autophagy at early time points after apoptosis induction is essential for RRV vFLIP to protect the cells from apoptosis. Our data is consistent with previous publications that explored both apoptosis and autophagy pathways. For example, two colon-cancer-derived cell lines, colon 26 and HT29, significantly underwent apoptosis after the combination treatment of 3-MA to inhibit autophagy and 5-FU to induce apoptosis [35]. Likewise, MCF-7, a breast cancer cell line, delayed apoptotic death following autophagy induction by nutrient starvation [34]. Inhibition of the expression of Beclin 1 and Atg7 stimulates apoptosis in DNA-damaged MCF-7 cells.

LC3-II was present at a relatively higher level in cells with RRV vFLIP expression than control cells even before apoptosis induction (Fig. 4A&B). We speculate that RRV vFLIP induces a basal level of autophagy to promptly respond to stress signals in order to promote cell survival. The greater accumulation of autophagosomes in HeLa-vFLIP stable cells is possibly due to increased formation or slower turnover rate. The data in Fig. 4 indicates that greater autophagosome formation might account for the increased level. Our data is consistent with previous observations that autophagy delays apoptosis of cancer cells. It has been reported that autophagy in MCF-7 cells due to nutrient starvation delays apoptotic death induced by camptothecin, a DNA-damaging compound [34]. Treatment with autopahgy inhibitors increased mitochondrial depolarization and caspase-9 activity, resulting in apoptosis. RRV vFLIP promotes autophagosome formation during early time points after apoptosis induction to prevent apoptotic cell death.

Although autophagy is considered a mechanism to enhance cell survival in adverse conditions, it is also classified as type II programmed cell death due to accumulation of autophagosomes in the cytoplasm under pathological conditions. It was reported that FLIP inhibits autophagic cell death induced by rapamycin by preventing Atg3 from binding and processing LC3 [20]. We found that RRV vFLIP is also able to inhibit rapamycin-induced autophagic cell death (unpublished observation), which is consistent with other vFLIPs described in the previous report. However, the enhancement of autophagosomes in HeLa-vFLIP stable cells after apoptosis induction indicates that a different mechanism is activated because vFLIP binding Atg3 inhibits autophagy. How the RRV vFLIP activates other mechanisms while avoiding the effect of interaction with Atg3 is not known. We speculate that RRV vFLIP activates autophagy through interaction with a cellular factor activated by apoptosis induction. The upregulation of MnSOD in HeLa-vFLIP stable cells is consistent with this speculation.

Since B lymphocytes are target cells in hosts in natural infection of RRV, BJAB cells were latently infected with RRV to verify the observation in HeLa cells with vFLIP expression. It has been reported that RRV latently and persistently infected immortalized B-cell lines [36]. The expression of the RRV vFLIP during latent phase was verified by real-time RT-PCR and Western blotting. Interestingly, BJAB cells with RRV latent infection resisted apoptosis induction. Moreover, when autophagy was inhibited with 3-MA or ammonium chloride, the BJAB cells with RRV infection lose the ability to escape from apoptosis. Suppression of vFLIP expression with siRNA leads to loss of the anti-apoptosis effect in BJAB-RRV, which indicates that vFLIP is possibly the viral gene responsible for the anti-apoptosis function. The data in BJAB cells is consistent with the observation in HeLa cells. There are other genes in RRV, such as vBcl-2, that inhibit autophagy and apoptosis. Our data indicates that these other genes may not be involved in the protection of cells from apoptosis induction via the autophagy pathway.

The exact mechanism for RRV vFLIP to enhance autophagosome formation during early apoptosis remains unclear. The autophagy pathway is implied to contribute in cell survival by cross talking with the apoptosis pathway. Mitochondria membrane permeabilization and cytochrome C release are a critical step during apoptotic cell death. However, when the damage in mitochondria is below the threshold required for apoptosis, the damaged mitochondria will be sequestered in autophagosomes. The autophagic process provides a source of metabolic energy in the form of ATP from damaged organelles and long-lived proteins. The effects of RRV vFLIP to enhance autophagy in apoptosis induction and inhibit rapamycin-induced autophagic cell death provide a great benefit to promote cell survival and prevent cell death. Further study on the interaction of RRV vFLIP with cellular factors is warranted and may yield informative data that can be extrapolated to assist the management of KSHV-associated malignancies.

In summary, our data show that RRV vFLIP inhibits apoptosis via the enhancement of the autophagy pathway to promote cell survival, while autophagic cell death is avoided. BJAB cells with RRV latent infection resists apoptosis induction and, when autophagy is inhibited, the apoptosis resistance disappears. Suppression of vFLIP expression in RRV-infected BJAB cells with siRNA also leads to loss of the apoptosis resistance, indicating that vFLIP-mediated activation of autophagy signaling protects the cells from apoptosis.

## Materials and Methods

### Cells and Viruses

Cell lines HeLa and HEK293 were maintained in Dulbecco’s modified Eagle’s medium (DMEM) supplemented with 10% fetal bovine serum (FBS). Transfection of the cells was accomplished with GeneExpresso 8000 (Lab Supply Mall, Gaithersburg, MD) as per the manufacturer’s instructions. Cell line RhF was a gift from B. Damania [8] and maintained in DMEM supplemented with 10% FBS. RRV26-95 was a gift from R.C. Desrosiers [36] and was propagated in RhF cells. BJAB cells were maintained in RPMI1640 medium supplemented with 10% FBS. Stable HeLa cells expressing CFP-LC3 have been described previously [28] and were maintained in DMEM supplemented with 10% FBS and G418 (Invitrogen, Carlsbad, CA) at 400 µg/ml.

### Plasmids

RRV ORF71 gene was amplified by PCR from RRV DNA isolated from culture supernatant of RRV-infected RhF cells. PCR was conducted with primers R71F2 and R71R3 (Table 1) that contain restriction sites for *Eco*RI and *Bam*HI, respectively, for directional cloning into a VenusN1 vector, as described previously [37]. PCR product with primers R71F2 and R71R2 was cloned into a VenusC1 vector. In VenusN1, ORF71 was cloned upstream of Venus; whereas in VenusC1, it was cloned downstream of Venus to confirm the vFLIP expression pattern. This resulted in two recombinant plasmids that expressed the vFLIP-Venus fusion protein after transfection.

**Table 1:** Primers used for ORF71 cloning and qPCR.

Primer^a^	Sequence (5′ to 3′)^b^	Plasmid or qPCR
R71F1	GC*GGATCC*TGTTCCC GCATAAGCGGTT	pGEX-3X
R71R1	GA*GAATTC*TTAACCGG GTGCGTTGGCG	
R71F2	GC*GAATTCC*ATGTTCC CGCATAAGCGGTT	VenusC1-R71
R71R2	GA*GGATCC*TTAACCGG GTGCGTTGGCGG	
R71F2	GC*GAATTCC*ATGTTCCC GCATAAGCGGTT	VenusN1-R71
R71R3	GA*GGATCCG*AACCGGG TGCGTTGGCGGC	
MnSOD-F1	GGAGAAGTACCAGGAGGCGT	qPCR
MnSOD-R1	TAGGGCTGAGGTTTGTCCAG	
Actin-F1	ATCGTGCGTGACATTAAG	qPCR
Actin-R1	ATTGCCAATGGTGATGAC	

a F, forward; R, reverse.

b Restriction sites of *Bam*HI, *Xho*I and *Eco*RI included in the primers are italicized.

The ORF71 gene was also amplified with primers R71F1 and R71R1 (Table 1) that contain restriction sites for *Bam*HI and *Eco*RI, respectively, and was cloned into pGEX-3X vector for prokaryotic protein expression and purification of vFLIP.

Construction of a CFP-LC3 plasmid has been previously described [25], [26]. Construction of mCherry-LC3 plasmid has been recently described [28].

### Protein Purification and Antibody Production

RRV vFLIP was expressed in BL21 *E. Coli* cells as a fusion protein of vFLIP-GST from a pGEX-3X-vFLIP plasmid with the induction of 1 mM IPTG (Promega, Madison, WI). The vFLIP-GST fusion protein was purified by B-PER GST Fusion Protein Purification Kits (Fisher Scientific, Pittsburgh, PA) according to the manufacturer’s instructions. Purified vFLIP-GST fusion protein was used to immunize rabbits for vFLIP antibody preparation (GenScript Corporation, Piscataway, NJ). Rabbit anti-vFLIP antibody was purified from the antiserum by affinity-purification with a CarboxyLink™ Kit (Fischer Scientific), which was used to covalently link purified vFLIP-GST fusion protein to agarose beads. The vFLIP antibody was verified by detection of vFLIP expressed in HeLa cells in Western blot analysis.

### Confocal Fluorescence Microscopy

Cells were seeded directly onto cell culture plates containing coverglass, incubated overnight, and transfected the next day. At 24 h post transfection, the coverglass was observed directly under confocal fluorescence microscopy or fixed with 1% paraformaldehyde and mounted onto a slide with anti-fade mounting solution containing 4′6′-diamidino-2-phenylindole (DAPI) (Invitrogen) before observation.

### Western Blot Analysis

Cells were transfected with either vFLIP expression plasmid or empty vector. At 24 h post-transfection, the cells were harvested with Laemmli sample buffer. Western blot analysis was conducted as previously described [38]. Briefly, the whole proteins in cell lysates were resolved in 12% polyacrylamide gel. The separated proteins were transferred to nitrocellulose membrane and probed with rabbit anti-vFLIP antibody. Any specific reaction was detected with goat anti-rabbit IgG conjugated with horseradish peroxidase (Sigma, St. Louis, MO) and revealed by the addition of chemiluminescence substrate. Chemiluminescence signal was collected by a ChemiDoc XRS imaging system (Bio-Rad Laboratories, Hercules, CA). Beta-tubulin was detected on the same blot membrane to normalize protein loading. Digital image acquisition and densitometry analyses were conducted by using Quantity One program (Version 4.6) (Bio-Rad). Similarly, expression of other proteins was detected with corresponding antibodies: GST (Rockland Immunochemicals Inc., Gilbertsville, PA), GFP, β-tubulin (Sigma), LC3 (Cell Signaling Technology, Danvers, MA), NF-κB p65, and PARP-1 (Santa Cruz Biotechnology, Santa Cruz, CA).

### Stable Expression of vFLIP in HeLa Cells

HeLa cells were transfected with VenusN1-vFLIP and the cells containing the plasmid were selected in medium supplemented with G418 (Invitrogen) at 400 µg/ml. Fluorescence-activated cell sorting was conducted to enrich cells with GFP expression. Sorted cells were plated and cultured to expand the cell population. Sorting and expansion were repeated three times to enrich and stabilize the cells with GFP expression. The expression of vFLIP was confirmed with Western blot analysis using rabbit anti-vFLIP antibody. The stable HeLa cells were stored in a liquid nitrogen container and used in this study.

### Apoptosis Induction

Tumor necrosis factor-α (TNF-α) (R&D Systems, Minneapolis, MN) and cycloheximide (Sigma) were added to cells at final concentrations of 50 ng/ml and 2.5 µg/ml, respectively, to induce apoptosis. The cells were harvested at different time points after apoptosis induction for RNA isolation or Western blot analysis, as indicated. Either TNF-α or cycloheximide alone at their respective concentrations used in this study cannot induce apoptosis. To inhibit the autophagosome formation step in autophagy, cells were treated with 3-methyladenine (3-MA) (Fisher) at a final concentration of 10 mM for one hour before apoptosis induction. To interrupt fusion and degradation of autophagosomes in lysosomes, cells were treated with ammonium chloride (NH4Cl) (Fisher) at a final concentration of 20 mM at 4, 6, and 8 h after apoptosis induction. At 10 h after apoptosis induction, the cells were harvested for Western blot analysis.

### Cell Viability Assay and Caspase Activity Detection

Cell viability was determined with CellTiter-Glo Luminescent Cell Viability Assay (Promega). Briefly, cells were cultured in a 96-well plate and CellTiter-Glo reagent was added and incubated for 10 minutes at room temperature. The luminescence signal was measured with a VICTOR^3^ Multilabel Counter (Perkin-Elmer, Waltham, MA). Relative percentages of luminescence intensity were calculated by comparison to mock-treated controls.

Activities of caspase-3, -7, -8, and -9 were detected with Caspase-Glo 3/7, Caspase-Glo 8, and Caspase-Glo 9 Assay kits (Promega). Caspase-Glo reagent was added to the cells and incubated for 30 minutes at room temperature. The luminescence signal was measured and relative percentages of luminescence intensity were calculated in comparison to controls.

For nutrient deprivation, the cells were seeded into 12-well culture plate and incubated overnight at 37°C. On the next day, the culture medium was replaced with Hank’s balanced salt solution (HBSS). The cells were either observed under a microscope or harvested for cell viability assay at 0, 24, and 48 h after the HBSS addition.

### Real-time PCR

Total RNA was isolated from cells lyzed in TRIzol reagent (Invitrogen). For real-time PCR analysis, RNA was first treated with RNase-free DNase (Promega) to remove carryover DNA from the RNA isolation procedure. Reverse transcription was carried out using AMV (avian myeloblastosis virus) reverse transcriptase and random hexamers (Promega). Real-time PCR primers that were used in this study were listed in Table 1. Real-time PCR with SYBR Green detection (Bio-Rad) was performed as previously described [39]. The actin transcript was also amplified from the same samples as an internal control for normalization. The gene expression levels in vFLIP-expressing cells were quantified via the 2^−ΔΔCT^ method in comparison with a vFLIP-negative control [40].

### NF-κB Reporter Assay

HeLa cells were co-transfected with a pGL4.32[*luc2P*/NF-κB-RE/Hygro] Vector (Promega) containing a NF-κB response element and either VenusN1-vFLIP or VenusN1 empty vector. VenusC1-vFLIP and empty VenusC1 vector were also used in this assay. Plasmid pRL-TK (Promega) was included as an internal control vector for all transfections. A prokaryotic vector pGEX-3X was used as a negative control. TNF-α was added to a few wells at a final concentration of 50 ng/ml at 24 h after transfection as a positive control for NF-κB activation. Dual-Glo Luciferase Assay System (Promega) was used to detect luciferase yield in the cells at 4 h after TNF-α addition following the manufacturer’s instructions. Relative folds of luciferase yields in the samples were calculated in comparison to a negative control.

### Subcellular Fractionation

Nuclear fraction was extracted from normal HeLa and HeLa-vFLIP stable cells using the CelLytic NuCLEAR Extraction Kit (Sigma). Cell collection, lysis, and subcellular fractionation were performed following the manufacturer’s instructions. The nuclear and cytoplasmic fractions were subjected to Western blot analysis. Antibodies against β-tubulin and PARP-1 were used to assess the fractionation efficiency.

### Preparation of siRNA and Transfection of BJAB Cells

The siRNA used in this study was designed with siRNA Target Designer (Version 1.6, Promega) and synthesized with the T7 RiboMA Express RNAi System (Promega). The sequence of siRNA against RRV ORF71 is 5′ GCTGGAGGCCGTGTTTCTC 3′. The efficiency of the siRNA against RRV ORF71 was tested in HEK293 cells transfected with VenusN1-vFLIP plasmid. Transfection of HEK293 cells with siRNA was accomplished with CodeBreaker siRNA Transfection Reagent (Promega) as instructed by the manufacturer. An irrelevant siRNA (5′ GAAATTACTGCACCTCGCC 3′) was used as a negative control. The BJAB cells were transfected with siRNA by using CodeBreaker siRNA Transfection Reagent. At 24 h post-transfection, the cells were harvested or treated for further analysis, as indicated in text.

### Statistical Analysis

A single factor pair-wise ANOVA statistical analysis was used to evaluate the significance in differences between levels of test parameters in the presence or absence of vFLIP. A two tailed *P*-value of less than 0.05 was considered significant.
